# Rifampicin resistant *Mycobacterium tuberculosis* in Vietnam, 2020–2022

**DOI:** 10.1016/j.jctube.2024.100431

**Published:** 2024-03-15

**Authors:** Hung Van Nguyen, Hoa Binh Nguyen, Doan Thu Ha, Dinh Thi Huong, Vu Ngoc Trung, Khieu Thi Thuy Ngoc, Tran Huyen Trang, Ha Vu Thi Ngoc, Tram Trinh Thi Bich, Trieu Le Pham Tien, Hanh Nguyen Hong, Phu Phan Trieu, Luong Kim Lan, Kim Lan, Ngo Ngoc Hue, Nguyen Thi Le Huong, Tran Le Thi Ngoc Thao, Nguyen Le Quang, Thu Do Dang Anh, Nguyễn Hữu Lân, Truong Van Vinh, Dang Thi Minh Ha, Phan Thuong Dat, Nguyen Phuc Hai, Derrick W. Crook, Nguyen Thuy Thuong Thuong, Nhung Viet Nguyen, Guy E. Thwaites, Timothy M. Walker

**Affiliations:** a National Lung Hospital, Hanoi, Viet Nam; b Vietnam National University, University of Medicine and Pharmacy, Viet Nam; c Oxford University Clinical Research Unit, Ho Chi Minh City, Viet Nam; d Pham Ngoc Thach Hospital, Ho Chi Minh City, Viet Nam; e Nuffield Department of Medicine, University of Oxford, Oxford, UK; f Centre for Tropical Medicine and Global Health, Nuffield Department of Medicine, University of Oxford, Oxford, UK

**Keywords:** Tuberculosis, Mdr, Vietnam

## Abstract

**Objective:**

We conducted a descriptive analysis of multi-drug resistant tuberculosis (MDR-TB) in Vietnam’s two largest cities, Hanoi and Ho Chi Minh city.

**Methods:**

All patients with rifampicin resistant tuberculosis were recruited from Hanoi and surrounding provinces between 2020 and 2022. Additional patients were recruited from Ho Chi Minh city over the same time period. Demographic data were recorded from all patients, and samples collected, cultured, whole genome sequenced and analysed for drug resistance mutations. Genomic susceptibility predictions were made on the basis of the World Health Organization’s catalogue of mutations in *Mycobacterium tuberculosis* associated with drug resistance, version 2. Comparisons were made against phenotypic drug susceptibility test results where these were available. Multivariable logistic regression was used to assess risk factors for previous episodes of tuberculosis.

**Results:**

*233/*265 sequenced isolates were of sufficient quality for analysis, 146 (63 %) from Ho Chi Minh City and 87 (37 %) from Hanoi. 198 (85 %) were lineage 2, 20 (9 %) were lineage 4, and 15 (6 %) were lineage 1. 17/211 (8 %) for whom HIV status was known were infected, and 109/214 (51 %) patients had had a previous episode of tuberculosis. The main risk factor for a previous episode was HIV infection (odds ratio 5.1 (95 % confidence interval 1.3–20.0); p = 0.021). Sensitivity for predicting first-line drug resistance from whole genome sequencing data was over 90 %, with the exception of pyrazinamide (85 %). For moxifloxacin and amikacin it was 50 % or less. Among rifampicin-resistant isolates, prevalence of resistance to each non-first-line drug was < 20 %.

**Conclusions:**

Drug resistance among most MDR-TB strains in Vietnam’s two largest cities is confined largely to first-line drugs. Living with HIV is the main risk factor among patients with MDR-TB for having had a previous episode of tuberculosis.

## 1 Background

After a brief interlude driven by SARS-CoV-2, tuberculosis is once again the most deadly global pathogen, killing more people each year than any other. Rifampicin has been the most effective anti-tuberculosis drug for over half a century, but ∼ 5 % of all circulating strains are now thought to be resistant. Whilst this remains a relatively small proportion, it still accounts for almost 500,000 people who develop rifampicin resistant disease annually [1].

According to the World Health Organization’s (WHO) estimates, Vietnam is 11th among countries with the largest number of people suffering from tuberculosis, with over 100,000 cases in 2022 [1]. Rifampicin resistant strains, often referred to as multi-drug resistant (MDR) strains as these are commonly also resistant to isoniazid, account for about 4 % of diagnoses. Notifications of MDR-TB have increased with wider access to MTB/RIF Xpert over the past decade. True incidence of MDR-TB may be higher still [2].

Providing rapid access to quality diagnostic services is a key goal for national tuberculosis programmes. Although MTB/RIF Xpert, and now Xpert Ultra, have provided much needed diagnostic capacity, they provide no drug-susceptibility information for the majority of anti-tuberculosis drugs [3]. In the presence of rifampicin resistance, drug susceptibility testing to other drugs is key to optimising patients’ chances of relapse free cure.

Culture based phenotypic testing remains slow, expensive and demanding of technical expertise. It is understandably not widely available in many low and middle-income countries [4]. Progress is more likely to be made with molecular approaches, with the WHO recently recommending targeted next generation sequencing (tNGS) as a diagnostic option for both rifampicin susceptible and rifampicin resistant tuberculosis [5]. The diagnostic accuracy of these platforms depends much on the catalogue of resistance mutations used to predict susceptibility from the identified genetic mutations. To this end WHO has also just released version 2 of its catalogue of mutations associated with drug resistance in *Mycobacterium tuberculosis*, which will serve not only as a reference to diagnostic platform manufacturers, but can also be used directly by national programmes or researchers who sequence isolates [6].

Here we conduct an analysis of rifampicin resistant tuberculosis in Vietnam. We explore characteristics of patients suffering from the disease, and use the WHO catalogue version 2 to describe genotypic patterns of drug resistance among MDR-TB isolates from Vietnam’s two largest cities; Hanoi and Ho Chi Minh City. Where availability of phenotypic susceptibility data allows, we attempt a validation of the catalogue in our setting.

## 2 Methods

### 2.1 Sampling frame

We aimed to prospectively collect all *M. tuberculosis* isolates testing resistant to rifampicin by Xpert MTB/RIF from adult patients (≥18 years old) in Hanoi from 2020. Patients came both from the city itself and surrounding provinces. We expected to recruit 400 patients but the SARS-CoV-2 pandemic slowed down recruitment and sample collection and a decision was made to supplement the collection from Hanoi with isolates from a parallel study of all patients with MDR-TB residing in Ho Chi Minh City. The isolates from Ho Chi Minh city were chosen on a convenience basis, focussing on those already in culture and for whom clinical data were already entered into the research database. We included only one sample per patient.

### 2.2 Sequencing and drug susceptibility testing

All samples were cultured in liquid culture (Mycobacterial Growth Indicator Tubes, MGIT, Becton Dickinson) and then on solid media (Löwenstein Jensen slopes). Isolates from Ho Chi Minh City were shipped to Hanoi where DNA was extracted using bead beating and AMPure XP beads purification. Whole genomes sequencing was performed for all isolates in Hanoi using an Illumina MiniSeq platform. FASTQ files were analysed using CRyPTIC’s clockwork pipeline (https://github.com/iqbal-lab-org/clockwork) [7]. Drug susceptibility predictions were made using version 2 of the WHO catalogue of mutations in *M. tuberculosis* associated with drug resistance [6]. Phenotypic drug susceptibility testing was performed in Hanoi using the proportion method in MGIT. Critical concentrations are listed in supplementary table S1.

### 2.3 Statistics

Sensitivity and specificity were calculated in two ways. Firstly, any strain with a resistance mutation was considered genotypically resistant and anything else genotypically susceptible. Secondly, a distinction was made on the one hand between strains with no mutations, or just with mutations consistent with susceptibility, and on the other hand strains with no resistance mutations but with mutations of ‘uncertain significance’. The latter were predicted as ‘U’ (unknown) and excluded from the sensitivity and specificity calculation. Multivariable logistic regression was used to explore risk factors for having had previous episodes of tuberculosis. Chi-squared tests were used elsewhere. All analyses were performed in STATA 18.

### 2.4 Ehical approval

The study was approved by the Institutional Review Board of the Ministry of Health, Hanoi, Vietnam (reference 3543/QĐ-BYT), and by the Oxford University Tropical Research Ethics Committee (OxTREC, reference 560–20), UK. The data generated in Ho Chi Minh City was from a study approved by Pham Ngoc Thach hospital ethics committee (643/PNT-HĐĐĐ) in Vietnam, and by OxTREC (reference 51–19) in the UK.

### 2.5 Data availability

Sequencing data in the form of FASTQ files are available under the project number PRJNA1044389 in EBI / NCBI.

## 3 Results

### 3.1 Study population

400 isolates were collected but only 265 were sequenced due to a limitation in resources within the study’s permitted time frame. Of those sequenced, 30 were of insufficient quality for further analysis, and two were not identified as *M. tuberculosis* from the WGS data. Of the 233 remaining strains, 146 (63 %) were from Ho Chi Minh City and 87 (37 %) from Hanoi. Of those sequenced, 198 (85 %) were lineage 2, 20 (9 %) were lineage 4, and 15 (6 %) were lineage 1. There was no difference in the lineage distribution between the two cities (p = 0.7 on chi^2^ test; supplementary table S2). Median patient age was 45 (IQR 34 to 54). 184 (79 %) were male and 49 (21 %) were female.

17/211 (8 %) for whom HIV status was known were infected, as were 15/216 (7 %) with an hepatitis B result, and 4/216 (2 %) with an hepatitis C result. Among patients for whom the variable was recorded, 112/213 (53 %) were either smokers or ex-smokers, and 130/214 (61 %) professed to never drinking alcohol. 109/214 (51 %) had had a previous episode of tuberculosis, of whom 17 had had more than one.

In a multivariable logistic regression model, the main risk factor for having had a previous episode of tuberculosis was HIV infection (odds ratio (OR) 5.1 (95 % confidence interval 1.3–20.0); p = 0.021), after adjusting for age, sex, diabetes, cigarette smoking and the city of residence (Table 1). Living in Ho Chi Minh city was a borderline risk factor (OR 2.1 (95 % confidence interval 1.0–4.2); p = 0.045).

**Table 1:** Risk factors among patients with MDR-TB for having had a previous episode of tuberculosis. Odds ratios derived from a multivariable logistic regression model. Patients for whom there was missing data for one or more variables used by the model were dropped from this analysis, explaining why some totals are smaller than in the complete dataset.

		First episode of TB	Previous episode of TB	Odds ratio	95 % confidence interval	p-value
Sex	Male	70	83	1.3	0.6–3.4	0.42
	Female	29	15			
HIV infection	Yes	3	13	5.1	1.3–20.0	0.02
	No	96	85			
Ever smoked	Yes	40	64	1.8	0.8–4.5	0.13
	No	59	34			
City of residence	Ho Chi Minh City	62	73	2.1	1.0–4.2	0.045
	Hanoi	37	25			
Diabetes	Yes	25	27	0.7	0.3–1.5	0.39
	No	74	71			
Age	(in years)			1.05	1.0–1.08	0.001

### 3.2 Genotypic drug susceptibility predictions

Even though an inclusion criterion for this study was rifampicin resistance, 17 strains had no genotypic evidence of rifampicin resistance on WGS analysis. Of these, 3 were phenotypically susceptible, 6 were phenotypically resistant and 8 had no phenotypic result. Ten had synonymous mutations in rpoB, but none of those could plausibly have led to a false positive MTB/RIF Xpert result as all were outside of the rifampicin resistance determining region.

Table 2 summarises the sensitivity and specificity of the genomic predictions among strains and drugs for which phenotypic drug susceptibility testing results were available. Where predictions were made for all isolates, sensitivity for isoniazid, rifampicin, ethambutol, streptomycin all exceeded 90 %. For moxifloxacin and amikacin, where the number of phenotypically resistant isolates were far fewer, sensitivity was much lower, at around 50 %. Specificity was low for rifampicin and ethambutol, but of 23 mutations resulting in apparent false positive results for rifampicin, 19 were at codons 430, 435, 445 and 452, all of which are associated with borderline MICs. Of 32 mutations resulting in apparent false positive results for ethambutol, 22 were at codon 306, and 5 at 406, both of which are also associated with modest elevations in the MIC and frequently associated with phenotypic error (Table 3) [8].

**Table 2:** Genotypic drug susceptibility predictions compared to phenotypic drug susceptibility test results. Predictions are derived from WGS data and are based on the WHO catalogue of drug resistance mutations, version 2. Sensitivity and specificity are computed once for all predictions, and once excluding strains that didn’t have a resistance associated mutation, but did have a mutation of ‘uncertain significance’.

	Phenotypically S			Phenotypically R			All ('U's count as 'S')		Excluding 'U's		
	Geno S	Geno R	Geno U	Geno S	Geno R	Geno U	Sensitivity	Specificity	Sensitivity	Specificity	Number U (%)
Isoniazid	5 (35.7)	0 (0)	9 (64.3)	4 (2.6)	150 (96.2)	2 (1.3)	96	100	97	100	11 (6)
Rifampicin	2 (7.7)	23 (88.5)	1 (3.8)	3 (2.1)	138 (95.8)	3 (2.1)	96	12	98	8	4 (2)
Ethambutol	5 (5)	32 (32)	63 (63)	1 (1.4)	65 (92.9)	4 (5.7)	93	68	98	14	67 (39)
Pyrazinamide	14 (20.6)	1 (1.5)	53 (77.9)	2 (2.2)	77 (84.6)	12 (13.2)	85	99	97	93	65 (41)
Moxifloxacin	123 (84.2)	1 (0.7)	22 (15.1)	10 (41.7)	11 (45.8)	3 (12.5)	46	99	52	99	25 (15)
Amikacin	124 (74.7)	0 (0)	42 (25.3)	2 (50)	2 (50)	0 (0)	50	100	50	100	42 (25)
Streptomycin	5 (14.7)	8 (23.5)	21 (61.8)	7 (5.1)	125 (91.9)	4 (2.9)	92	76	95	38	25 (15)

**Table 3:** Mutations resulting in apparent false positive predictions for rifampicin and ethambutol.

Rifampicin	n
rpoB_p.1309delAAC	1
rpoB_p.Asp435Tyr	3
rpoB_p.His445Asn	4
rpoB_p.His445Gly	1
rpoB_p.His445Ser	2
rpoB_p.Leu430Pro	4
rpoB_p.Leu452Pro	5
rpoB_p.Ser450Leu	1
rpoB_p.Ser450Val	1
rpoB_p.Thr444Thr	1
Ethambutol	
embB_p.Asp328Tyr	1
embB_p.Asp354Ala	1
embB_p.Gln497Arg	3
embB_p.Gly406Asp	3
embB_p.Gly406Ser	2
embB_p.Met306Ile	10
embB_p.Met306Val	12

Where predictions were made only for isolates for which the catalogue described all observed mutations either as predictive of resistance or consistent with susceptibility, and non as of ‘uncertain significance’, there was an improvement in sensitivity but a deterioration in specificity. Sensitivity for first-line drugs was 97 % or greater, and 95 % for streptomycin. No real change was seen for moxifloxacin and amikacin. Specificity declined most dramatically for ethambutol and streptomycin, in both cases due to the magnification in the relative contribution of a few ‘false’ positive mutations after a large number of phenotypically susceptible isolates with mutations of uncertain significance were removed.

### 3.3 Drug resistant mutations seen

The drug-resistance mutations from the WHO catalogue which are seen in MDR-TB in our Vietnamese isolates are listed in supplementary table S3. *katG_p.Ser315Thr* and *fabG1_c.-15C > T* accounted for 95 % of isoniazid resistance mutations, and *rpoB_p.Ser450Leu* accounted for 51 % of rifampicin mutations. Although the two previously characterised resistance mutations that lie outside of the rifampicin resistance determining region (RRDR) were each seen once (*rpoB_p.Ile491Phe* and *rpoB_p.Val170Phe*), these both co-occurred with RRDR mutations and so will have been identifiable by MTB/RIF Xpert. *embB* codon 306 mutations accounted for 77 % of ethambutol resistance mutations; *rpsL_p.Lys43Arg* and *rpsL_p.Lys88Arg* for 78 % of streptomycin resistance mutations; *gyrA* codon 94 mutations for 81 % of moxifloxacin resistance; and *fabG1_c.-17G > T*, *- fabG1_c.15C > T*, and *fabG1_c.609G > A* for 73 % of ethionamide resistance mutations. No bedaquiline or linezolid resistance mutations were seen, but 5 *fbiB* mutations associated with cross resistance to clofazimine and delamanid were observed (*fbiB_p.416delGG; fbiB_p.419delT; fbiB_p.395delTG; fbiB_p.1508delA; fbiB_p.402delCGGGCTGCGCG*), along with 2 mutations in *ddn* that are associated with resistance to just the latter (*ddn_p.Trp88!; ddn_p.Leu49Pro*). No associated phenotypes were available for these drugs.

### 3.4 Antibiotic profiles

**Fig. 1:**
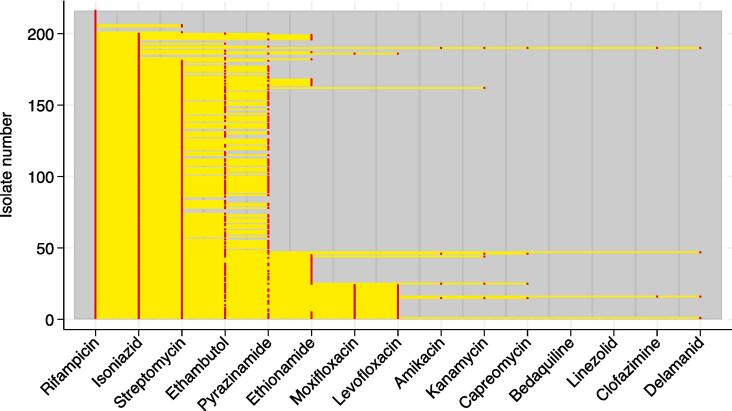
Genotypic antibiotic profile predictions for all isolates identified as rifampicin resistant by WGS. Red markers indicate predicted drug resistance whereas the absence of a red marker indicates that no resistance mutations are present in the strain. Yellow lines connect red markers for each strain. (For interpretation of the references to colour in this figure legend, the reader is referred to the web version of this article.)

Finally, genotypic drug susceptibility profiles were analysed to explore patterns of resistance to other drugs among genotypically rifampicin resistant strains (Fig. 1; supplementary table S4). 200/216 (93 %) were resistant to isoniazid, of which 126 (63 %) were also ethambutol resistant. Of those 126, 80 (63 %) were resistant to pyrazinamide. Only 25/216 (12 %) isolates were resistant to Moxifloxacin, of which 12 (48 %) were resistant to all 4 first line drugs. All moxifloxacin resistant isolates were also isoniazid resistant. Resistance to injectables, new and re-purposed drugs was rare. Full genotypic and phenotypic profiles linked to sequencing read archive accession numbers can be found in supplementary table S5.

## 4 Discussion

Understanding the local epidemiology of drug resistance patterns is key to any national tuberculosis programme being able to make informed decisions when planning treatment services and diagnostic capacity. Here we describe patient and isolate characteristics among a prospectively recruited cohort of patients and their rifampicin resistant isolates collected from Hanoi and Ho Chi Minh City in Vietnam.

We found a plurality of men among the rifampicin resistant cases, with over half being smokers and over half having had a previous episode of tuberculosis. The main risk factor for having had a previous episode was having a diagnosis of HIV infection, although patients from Ho Chi Minh city were also marginally more at risk. We had no data on whether past episodes of tuberculosis were rifampicin resistant or not.

We find that genotypic resistance to streptomycin, ethambutol and pyrazinamide is very common among strains resistant to rifampicin and isoniazid, but that genotypic resistance to other anti-tuberculosis drugs is much less common in these cities, as has also been reported elsewhere in South-East Asia [9]. There was very little genotypic resistance to new or repurposed drugs. For first-line drugs and streptomycin, sensitivity of genomic predictions was high but specificity was low for rifampicin, ethambutol and streptomycin. This was a consequence of well-established resistance associated mutations that confer only modest increases in the MIC and consequently increase the probability of phenotypic error due to the proximity of the MIC to the critical concentration [8], [10]. In the case of streptomycin, it was largely loss of function mutations which the WHO expert rules predict to confer resistance that were responsible for the poor specificity [6]. With regards to the low sensitivity for moxifloxacin, it is not uncommon for fluoroquinolone resistance to be conferred by low frequency alleles which may not have been detected here [11]. tNGS, which often produces a higher depth of coverage, may be better tool when it comes to detecting those.

The consequence of making predictions only for strains with mutations that were listed as resistance associated or consistent with susceptibility in the WHO catalogue was a modest improvement in sensitivity but for ethambutol and streptomycin a dramatic reduction in specificity. This was because most ‘U’ mutations relevant to these drugs were largely in phenotypically susceptible strains. By removing otherwise susceptible looking strains that contain ‘U’ mutations, we enriched strongly for strains with genuine resistance mutations where the phenotype was wrong. When doing such analyses, data contributing to specificity results should thus always be scrutinised carefully. When it comes to phenotypic DST, not all that glisters is gold standard.

Version 2 of the WHO catalogue will have to be more thoroughly validated with a larger dataset, and one that also includes ample susceptible samples. However, this relatively small-scale analysis underscores previously observations that most drug resistance is caused by a small number of mutations that are found widely around the world [10]. Although one or two mutations have been identified outside of Vietnam that are more geographically concentrated [12], WHO recommended molecular diagnostic platforms would be expected to perform well in Vietnam based on our results.

There are a number of limitations to our study. We did not have phenotypic DST results for many drugs, including any of the new or repurposed drugs. There may thus be drug resistance among our isolates that we have not described. As we only intended to look at rifampicin resistant strains, a more meaningful validation of version 2 of the WHO catalogue was not possible. There were evidently some sample handling errors with the relatively high number of isolates that were not resistant to rifampicin. Although lineage 1 has been linked to higher MICs to pyrazinamide and pretomanid [13], and although lineage 1 is abundant in Asia, only 6 % of our isolates were lineage 1 and we were unable to comment further on this. We lacked data on the drug resistance patterns of previous episodes of TB, or indeed the drugs that were prescribed at the time, and thus our analysis of risk factors for having had a previous episode is by no means conclusive.

Nevertheless, this study provides a helpful overview of the characteristics of patients and their rifampicin resistant *M. tuberculosis* strains in Vietnam. We observe similar drug resistance conferring mutations to those seen in other countries, reinforcing confidence in existing WHO recommended molecular assays which probe these mutations, and identify relatively little circulating drug resistance outside of first line drugs. And we provide the first independent validation of version 2 of the WHO’s catalogue of mutations associated with drugs resistance. Further validations will be needed.

## 5 Conclusion

Based on our sampling frame, drug resistance among most MDR-TB strains in Vietnam’s two largest cities is still relatively confined to first-line drugs. The mutations underlying drug resistance are of a familiar pattern seen elsewhere. Living with HIV is the main risk factor we identified for having had a previous episode of tuberculosis, despite the relatively low incidence of HIV/TB co-infection [14].

## CRediT authorship contribution statement

**Hung Van Nguyen:** Writing – review & editing, Resources, Project administration, Methodology, Investigation, Funding acquisition, Data curation, Conceptualization. **Hoa Binh Nguyen:** Writing – review & editing, Supervision, Resources, Funding acquisition, Data curation, Conceptualization. **Doan Thu Ha:** . **Dinh Thi Huong:** . **Vu Ngoc Trung:** . **Khieu Thi Thuy Ngoc:** . **Tran Huyen Trang:** Writing – review & editing, Data curation. **Ha Vu Thi Ngoc:** Writing – review & editing, Project administration, Methodology, Formal analysis, Data curation. **Tram Trinh Thi Bich:** Writing – review & editing, Supervision, Project administration, Methodology, Investigation, Formal analysis, Data curation. **Trieu Le Pham Tien:** Data curation, Methodology. **Hanh Nguyen Hong:** . **Phu Phan Trieu:** Writing – review & editing, Resources, Methodology, Data curation. **Luong Kim Lan:** . **Kim Lan:** Writing – review & editing, Data curation. **Ngo Ngoc Hue:** . **Nguyen Thi Le Huong:** . **Tran Le Thi Ngoc Thao:** . **Nguyen Le Quang:** . **Thu Do Dang Anh:** Writing – review & editing, Data curation. **Nguyễn Hữu Lân:** . **Truong Van Vinh:** Writing – review & editing, Resources, Project administration. **Dang Thi Minh Ha:** . **Phan Thuong Dat:** Writing – review & editing, Resources, Project administration, Data curation. **Nguyen Phuc Hai:** . **Derrick W. Crook:** Writing – review & editing, Supervision, Resources, Project administration, Methodology, Funding acquisition, Conceptualization. **Nguyen Thuy Thuong Thuong:** . **Nhung Viet Nguyen:** Writing – review & editing, Resources, Project administration, Methodology, Funding acquisition, Conceptualization. **Guy E. Thwaites:** Writing – review & editing, Supervision, Resources, Project administration, Methodology, Investigation, Funding acquisition, Conceptualization. **Timothy M. Walker:** Writing – original draft, Supervision, Software, Project administration, Methodology, Investigation, Funding acquisition, Formal analysis, Data curation, Conceptualization.

## Declaration of competing interest

The authors declare that they have no known competing financial interests or personal relationships that could have appeared to influence the work reported in this paper.
